# Formation of a P_16_^2–^ Ink from Elemental Red Phosphorus in a Thiol–Amine Mixture

**DOI:** 10.1021/acs.inorgchem.3c00370

**Published:** 2023-04-11

**Authors:** Marissa J. Strumolo, Dmitry B. Eremin, Shuai Wang, Carlos Mora Perez, Oleg V. Prezhdo, Joshua S. Figueroa, Richard L. Brutchey

**Affiliations:** † Department of Chemistry, University of Southern California, Los Angeles, California 90089, United States; ‡ The Bridge@USC, University of Southern California, Los Angeles, California 90089, United States; § Department of Chemistry and Biochemistry, University of California—San Diego, La Jolla, California 92093, United States

## Abstract

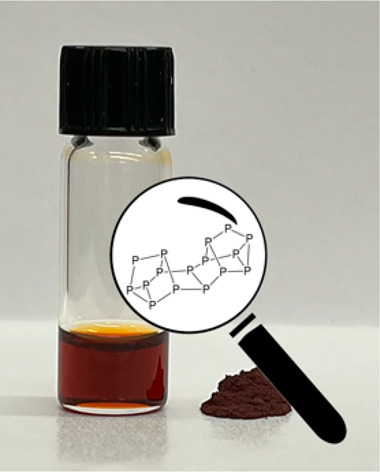

A P_16_^2–^ polyphosphide dianion ink was produced by the reaction of red phosphorus with a binary thiol–amine mixture of ethanethiol (ET) and ethylenediamine (en). The polyphosphide was identified by solution ^31^P NMR spectroscopy and electrospray ionization mass spectrometry. This solute was compared to the reaction products of white phosphorus (P_4_) and other elemental pnictides in the same solvent system. The reaction of P_4_ with ET and en gives the same P_16_^2–^ polyphosphide; however, the easier handling and lower reactivity of red phosphorus highlights the novelty of that reaction. Elemental arsenic and antimony both give mononuclear pnictogen–sulfide–thiolate complexes upon reaction with ET and en under otherwise identical conditions, with this difference likely resulting from the greater covalency and tendency of phosphorus to form P–P bonds.

## Abstract 2

A new method of forming the P_16_^2−^ polyphosphide dianion is reported by reacting red phosphorus with a combination of dithiol and diamine.

Phosphorus has long been an element of interest because it has several allotropes of varying reactivity, and it can form homonuclear P–P bonds similarly to carbon.^1^ Polyphosphides are anionic, multinuclear, trivalent phosphorus clusters that contain different skeletal arrangements, including rings and cages.^2^ In this way, polyphosphides represent “element-near” phosphorus species that bridge the gap between molecular and solid-state compounds. Polyphosphides exhibit interesting reactivity (e.g., partial protonation yielding polyphosphane intermediates), in addition to possessing novel optoelectronic and magnetic properties.^3−5^ Unfortunately, limitations still exist with the synthesis of polyphosphides themselves.

Traditionally, polyphosphides have been synthesized using one of two methods: solution-based reactions of white phosphorus (P_4_) or solid-state reactions of red phosphorus. The reaction of alkali metals with P_4_ can be carried out in liquid ammonia or polar organic solvents. These reactions can selectively yield various polyphosphides depending on the ratio of the alkali metal to phosphorus and the solvent.^6^ Alternatively, polyphosphides can be synthesized via a nucleophilic attack of P_4_ by LiPH_2_ or LiP(TMS)_2_ in coordinating solvents.^7,8^ Despite the advantageous solubility and reactivity of P_4_, it is hazardous, making P_4_ a nonideal elemental phosphorus source.^9^ Red phosphorus would therefore seem to be a preferred alternative as an air-stable phosphorus allotrope; however, it is insoluble in water and most organic solvents.^10^ As such, reactions between red phosphorus and reducing agents have typically been confined to the solid state. These reactions are highly exothermic, and local overheating can lead to explosions.^5^ An alternative approach was developed to synthesize polyphosphides from the nucleophilic activation of red phosphorus in various organic solvents using KOEt.^11^ While this method facilitates the solution-phase formation of polyphosphides from red phosphorus, KOEt reacts with water and can be self-heating in air.

We reported on the ability of a thiol–amine mixture to dissolve typically insoluble gray selenium and tellurium to produce soluble polychalcogenide inks.^12^ While binary oxides and chalcogenides of the heavier pnictogens can be dissolved using this method,^13^ the dissolution of elemental phosphorus is heretofore unexplored. In this work, red phosphorus was successfully dissolved in a mixture of ethanethiol (ET) and ethylenediamine (en). The P_16_^2–^ polyphosphide dianion ink produced in this reaction was identified by solution ^31^P NMR and negative-ion-mode electrospray ionization mass spectrometry [ESI-(−)MS] and compared to and contrasted with the products resulting from P_4_ and other elemental pnictogens.

The dissolution reaction of red phosphorus (20 mg/mL) was performed in an air-free environment with mild heating (55 °C) for 48 h. Neat en dissolves red phosphorus, consistent with prior reports,^14^ at a thermogravimetrically determined solubility limit of ca. 5 mg/mL. Neat ET is not able to dissolve any red phosphorus. When ET and en are combined, the nucleophilic character of the mixture is much greater, due to some degree of deprotonation of the thiol by the amine. Thiolate formation is experimentally supported by Fourier transform infrared (FT-IR) spectroscopy, with the clear loss of the weak sulfhydryl stretch at ν(S–H) 2560 cm^–1^ upon the addition of en to ET (Figure S1). Likewise, increased ion formation in the binary mixture is evidenced by a 200× increase in the electrolytic conductivity upon the addition of en to ET. This is further supported by the dissolution of red phosphorus. Using a 1:4 (v/v) thiol–amine mixture, the solubility of red phosphorus was doubled, resulting in a thermogravimetrically determined solubility of ca. 10 mg/mL after 48 h. In the reaction with red phosphorus, ET is oxidized to form sulfinic and sulfonic acids, which points to the potential role of ET as a reducing agent. ESI-(−)MS of neat ET shows trace amounts of [C_2_H_5_SO_2_]^−^ and [C_2_H_5_SO_3_]^−^ at *m*/*z* 91.9 and 107.9, respectively, with a significant increase in the abundance of these species in the reaction mixture with red phosphorus. After filtration, the reaction results in a free-flowing solution that does not scatter light. The experimental and calculated UV–vis spectra of the dissolved red phosphorus are consistent with previous literature for polyphosphides and show high absorbance in the near-UV region, leading to a deep-red color (Figure 1).^11^ The computational methodology and detailed analyses for our calculated spectrum are provided in the Supporting Information. Natural transition orbital pairs reveal the nature of the key electronic excitations, involving transitions from a localized π orbital near the periphery of the polyphosphide to a delocalized orbital near the center of the molecule (Supporting Information). Time studies using solution ^31^P NMR and ESI-(−)MS confirmed the formation of polyphosphide in as little as 3 h, which then peaks after 3 days (Figure S8).

**Figure 1:**
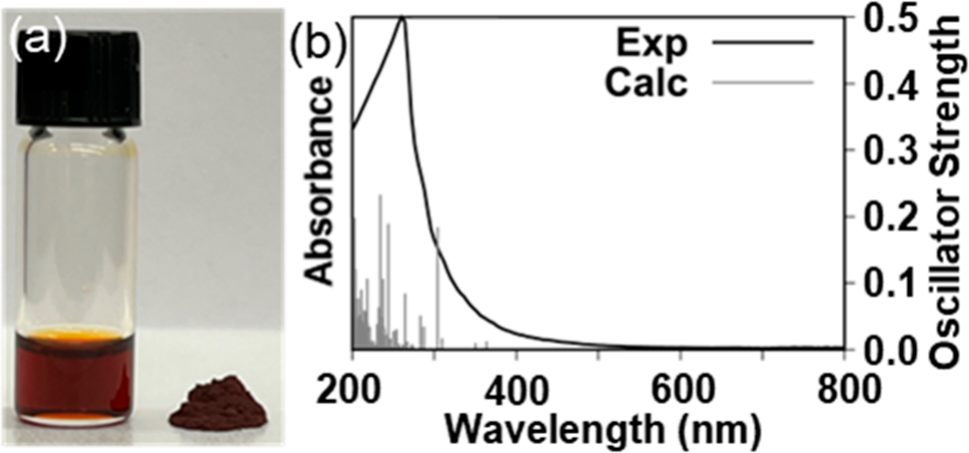
(a) Photograph of red phosphorus and a solution of dissolved red phosphorus in ET and en. (b) Absorption spectrum of dissolved red phosphorus in ET and en diluted in *N*,*N*-dimethylformamide (DMF) in black, with the calculated spectrum shown by gray sticks (oscillator strength, right scale). The solvent UV cutoff for DMF is 268 nm.

Solution ^31^P{^1^H} NMR gave a singlet that resonates at 114 ppm. Previous work has shown that P_4_ reacts with thiolates to form thiophosphites in this ^31^P chemical shift range;^15^ therefore, the singlet at 114 ppm was assigned to P(SEt)_3_. In addition, six multiplets are observed at −173, −132, −38, 3, 40, and 61 ppm. These multiplets correspond to the hexadecapolyphosphide anion (P_16_^2–^), where the corresponding countercation would be protonated en.^11^ The P_16_^2–^ polyphosphide has a conjunctophosphane skeleton that can be thought of as two P_7_ units that are coupled via a common P_2_ dumbbell, which gives six chemically distinct phosphorus atoms. The remaining singlets at −2.5, 3, and 11 ppm were not able to be assigned (Figure 2a); however, this chemical shift range is indicative of quaternary phosphonium species, phosphorus(V) oxo compounds, and di- and monoalkyl thiophosphites (i.e., HP(SEt)_2_ and H_2_P(SEt)^15,16^). The proton-coupled ^31^P NMR reveals that all three of the remaining peaks possess coupling to one or more hydrogen atoms (Figure S9). The lack of a signal in the positive-ion-mode ESI-(+)MS suggests that there are no quaternary phosphonium species in solution. Gas chromatography (GC)–MS revealed the presence of both HP(SEt)_2_ and H_2_P(SEt), which may account for two of the unknown peaks in the solution ^31^P NMR spectrum. Reactions performed at lower concentrations of red phosphorus (i.e., 5 mg/mL) returned the same ^31^P{^1^H} NMR spectrum, suggesting that the chemistry is not strongly concentration-dependent (Figure S10).

**Figure 2:**
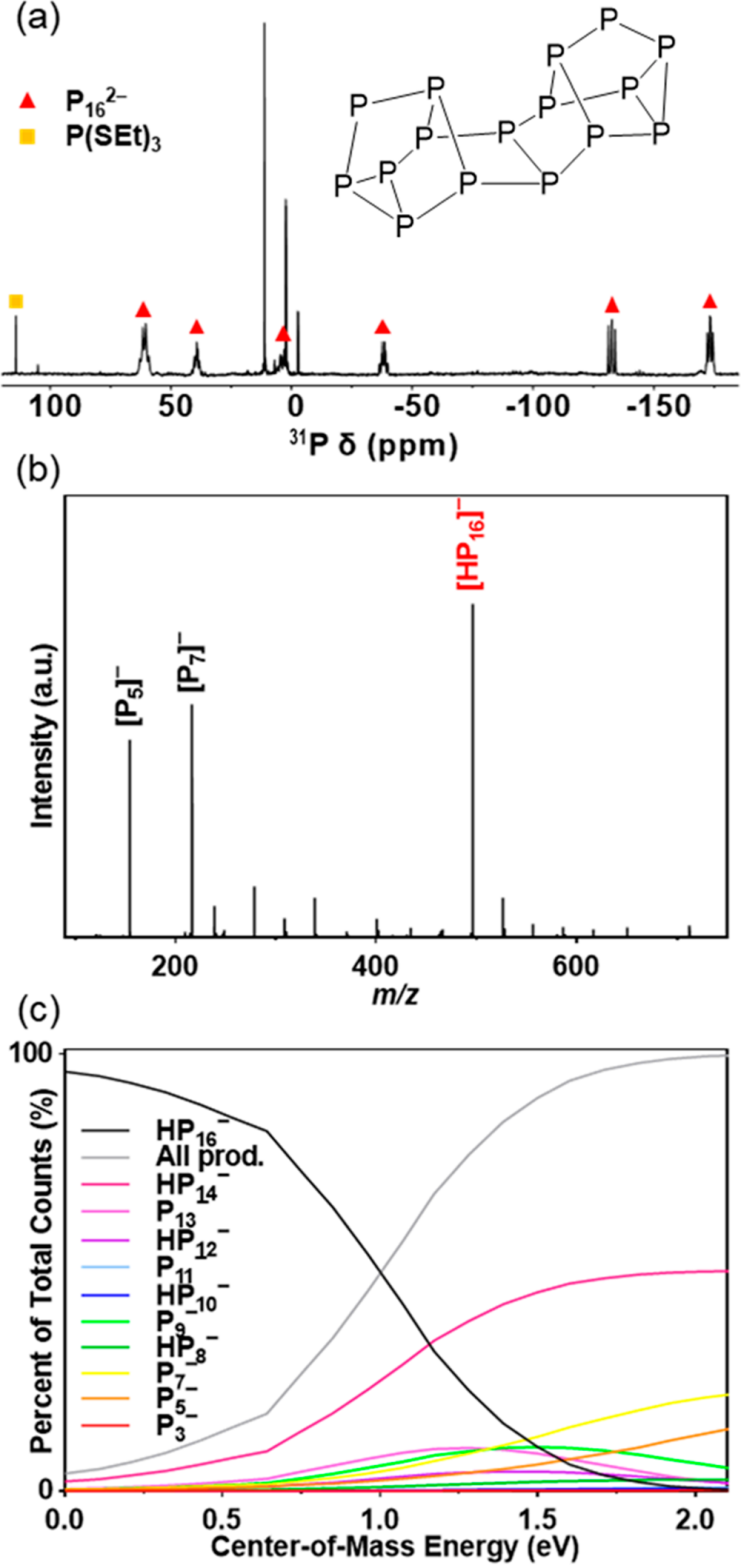
Identification of P_16_^2–^ from the reaction of red phosphorus with ET and en. (a) Solution ^31^P{^1^H} NMR spectrum of P_16_^2–^ and P(SEt)_3_, denoted by ▲ and ■, respectively, in acetonitrile-*d*_3_. The inset shows the structure of P_16_^2–^. (b) ESI-(−)MS of dissolved red phosphorus in ET and en, diluted 100× volumetrically in DMF. The major peak at *m*/*z* 496.6 is assigned as the parent ion [HP_16_]^−^. (c) Normalized CID data showing the fragmentation of the parent ion into smaller polyphosphide species.

The assignment of P_16_^2–^ was further established by high-resolution ESI-(−)MS (Figure 2b). The major peak in the mass spectrum at *m*/*z* 496.6 is identified as the parent [HP_16_]^−^ ion. Two other polyphosphide species, [P_7_]^−^ and [P_5_]^−^, are identified at *m*/*z* 216.8 and 154.9, respectively, as less abundant signals. Collision-induced dissociation (CID) shows that the parent [HP_16_]^−^ ion has reasonable stability, with a 50% survival yield of 1.17 eV (center-of-mass lab frame energy, *E*_CM_). The ions formed upon fragmentation range from [P_3_]^−^ to [HP_14_]^−^, where all odd-numbered polyphosphides are monoanions and all even-numbered polyphosphides are monoprotonated. After *E*_CM_ ∼ 2 eV, secondary fragmentation of the daughter ions led to the production of additional [P_7_]^−^ and [P_5_]^−^ (Figure 2c). The presence of these two ions in the initial spectrum likely results from partial fragmentation during the ionization and ion-transfer process. However, in solution, P_16_^2–^ is stable under inert atmosphere for over 1 week but reacts with air within hours. The appearance of resonances near 0 ppm by ^31^P NMR suggests the formation of phosphate species when the solution is exposed to air under ambient conditions. Full degradation of P_16_^2–^ occurs within 72 h after exposure to air (Figure S11).

The production of P(SEt)_3_ and P_16_^2–^ from the reaction of red phosphorus with ET and en is noteworthy when compared to previously reported reactions between red phosphorus and potassium monothiolates in dimethyl sulfoxide, which yielded dialkyl trithiophosphates.^17^ Shatruk and co-workers subsequently showed that changing the solvent to a 1:1 (v/v) mixture of tetrahydrofuran and dimethoxyethane resulted in the selective formation of P_16_^2–^ from the equimolar reaction of red phosphorus with various sodium monothiolates.^18^ The different product slate observed here with a dithiol and a diamine suggests a disproportionation reaction that proceeds with the presence of excess nucleophile,^6^ which is consistent with the conditions used in our reaction.

The reaction mixture was dried and annealed under flowing nitrogen to 320 °C. The sample was not annealed to higher temperatures because red phosphorus begins to volatilize over 300 °C.^19^ Annealing produced a red-orange material that is consistent with the recovery of highly amorphous red phosphorus. The powder X-ray diffraction (XRD) pattern of the recovered material shows three broad reflections at 2θ = 15.5, 32.5, and 53.0°, which match the XRD pattern of the as-purchased red phosphorus, with two more broad reflections that suggest an additional amorphous component. Energy-dispersive X-ray spectroscopy maps of the recovered material show the presence of phosphorus. The Raman spectrum of the recovered material showed very diffuse bands between 300 and 500 cm^–1^ that are consistent with the three Raman bands at ∼350, 390, and 460 cm^–1^ observed in the as-purchased red phosphorus (Figures S12 and S13).^19^ In addition, the Raman spectrum of the recovered material shows a strong band at ca. 560 cm^–1^ corresponding to ν(C–S) from residual thiol(ate).

P_4_ reacts with an analogous mixture of ET and en in a matter of minutes at room temperature. After the solution was heated to 55 °C for 48 h (i.e., identical to the reaction conditions for red phosphorus), the solution ^31^P{^1^H} NMR spectrum clearly shows the six peaks associated with P_16_^2–^ and four additional singlets at 3, 11, 20, and 28 ppm (Figure 3a). The two peaks resonating at 20 and 28 ppm had no proton coupling, but the other two at 3 and 11 ppm had strong proton coupling to one or more hydrogen atoms and are consistent with peaks observed from the reaction of red phosphorus (Figure S14). The dissolution of P_4_ in the same mixture of ET and en produces the P_16_^2–^ polyphosphide without the concomitant formation of P(SEt)_3_, as is observed with red phosphorus. This stands in contrast to the reaction of P_4_ and sodium thiolates in the presence of electrophilic CCl_4_, which yields P(SR)_3_ without polyphosphide formation.^20,21^ As expected, when this reaction mixture with P_4_ was dried and annealed under flowing nitrogen to 320 °C, the Raman spectrum of the resulting solid was consistent with that of highly amorphous red phosphorus (Figure S13).

**Figure 3:**
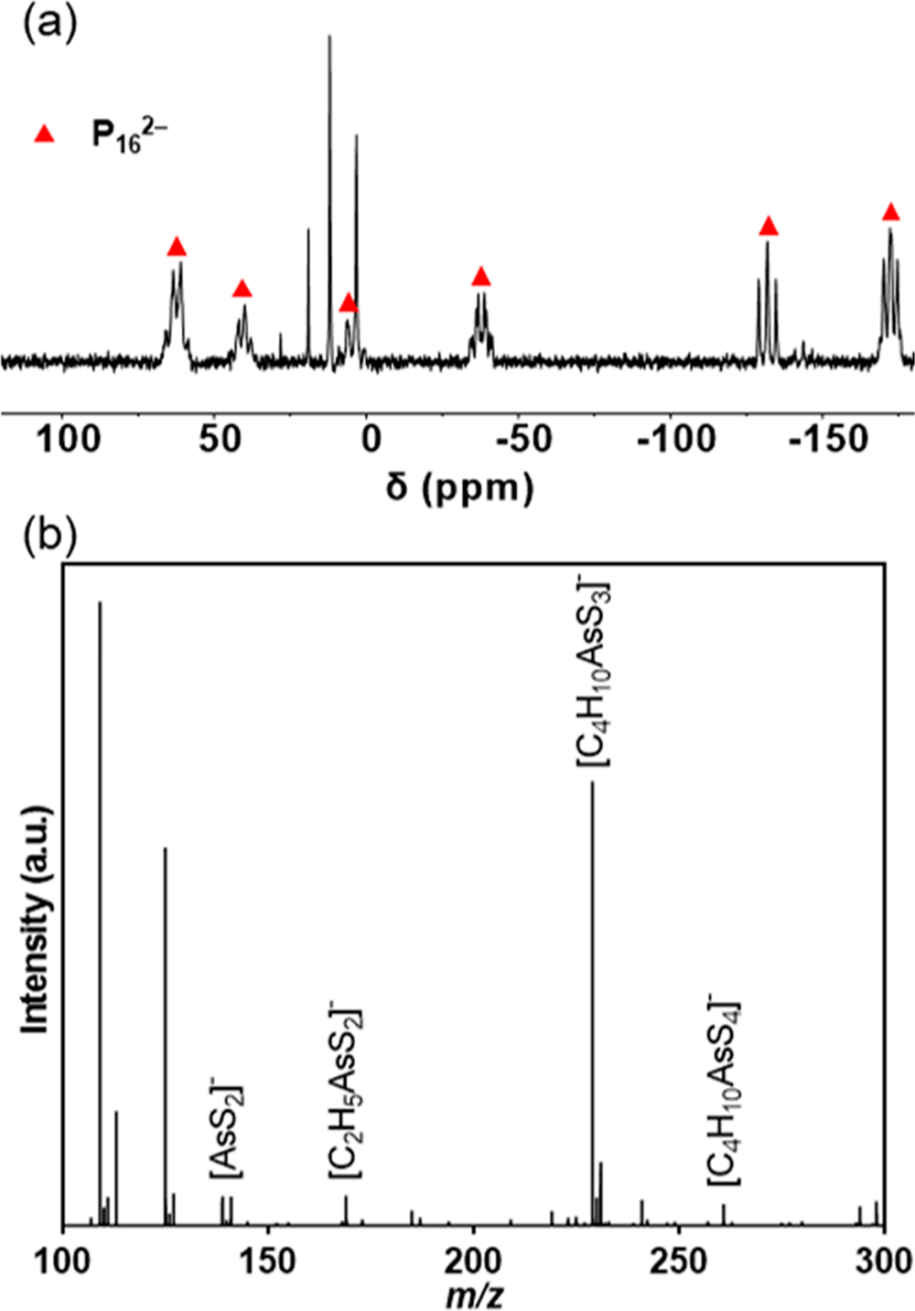
Identification of dissolution products for other pnictogens. (a) Solution ^31^P{^1^H} NMR spectrum of the reaction of P_4_ with ET and en, in acetonitrile-*d*_3_. The P_16_^2–^ product is denoted by ▲. (b) ESI-(−)MS of dissolved gray arsenic in ET and en, diluted 100× in DMF.

When elemental gray arsenic and antimony were combined with ET and en in an analogous fashion, the thermogravimetrically determined solubility was lower (<5 mg/mL). The reaction of gray arsenic resulted in a light-pink solution, and the elemental antimony resulted in a yellow solution. Analysis by ESI-(−)MS suggests that both elements react similarly to previous reports of Bi, Bi_2_O_3_, and Sb_2_S_3_ in thiol–amine solutions, in which solutes consist of pnictogen–sulfide–thiolate complexes.^22,23^ The major peak in the ESI-(−)MS spectrum of the gray arsenic solution appears at *m*/*z* 228.9 and is assigned to a complex in which arsenic exists as As(III) and is bound to two ethanethiolate ligands and a single sulfide ligand, giving the formula [C_4_H_10_AsS_3_]^−^. The presence of heavier arsenic complexes may indicate that the major peak is not the parent ion but the most stable fraction of the parent ion (Figure 3b). Similarly, while the major peak of the antimony solution appears at *m*/*z* 274.9, a second large peak appears at *m*/*z* 306.9. These peaks can be assigned as pnictogen–sulfide–thiolate complexes, giving the formulas [C_4_H_10_SbS_3_]^−^ and [C_4_H_10_SbS_4_]^−^, such that antimony exists in the Sb(III) and Sb(V) oxidation states, respectively. The position of the major peak at a lower *m*/*z* value than that of the largest ion may indicate that the parent peak is at *m*/*z* 306.9 but is unstable when ionized (Figure S15). The difference in reaction products between the heavier pnictogens and red phosphorus likely results from the greater covalency and tendency of phosphorus to form homonuclear P–P bonds.

In summary, we showed that a P_16_^2–^ ink is produced by reacting red phosphorus with a mixture of ET and en, using a combination of ^31^P NMR and ESI-(−)MS. The solution returns amorphous red phosphorus when dried and annealed. The same polyphosphide was also produced in an analogous reaction with P_4_. Given that some previously reported reactions between thiolates and red phosphorus or P_4_ give mononuclear phosphorus compounds, this suggests that polyphosphide formation results from the specific combination of thiol and amine used here and not the phosphorus precursor. Reaction of gray arsenic and antimony in the same thiol–amine mixture produced mononuclear pnictogen–sulfide–thiolate complexes by ESI-(−)MS, much like previous work with elemental bismuth.^23^ The synthesis of polyphosphides using red phosphorus in reagent combinations that also readily dissolve a host of other elemental sources and metals may enable further exploration and application of these species.

## Supporting Information Available

Experimental details, details of the TD-DFT calculations, FT-IR spectra of ET/en, GC–MS trace of the reaction mixture, additional ^31^P NMR spectra, and XRD patterns, EDX maps, and Raman spectra of recovered red phosphorus (PDF)
