# Supplementary material for: Formation of a P162– Ink from Elemental Red Phosphorus in a Thiol–Amine Mixture
Source: Inorg Chem. 2023 Apr 11;62(16):6197–201. doi: 10.1021/acs.inorgchem.3c00370 (PMC10131223; doi:10.1021/acs.inorgchem.3c00370)
Supplement: Supplementary file 1 — ic3c00370_si_001.pdf [file ic3c00370_si_001.pdf]

Supporting Information

Formation of a P_{16}^{2-} Ink from Elemental Red Phosphorus in a Thiol-Amine Mixture

Marissa J. Strumolo,[a] Dmitry B. Eremin,[a,b] Shuai Wang,[c] Carlos Mora Perez,[a] Oleg V. Prezhdo,*[a] Joshua S. Figueroa,*[c] and Richard L. Brutchey*[a]

[a] Department of Chemistry, University of Southern California, Los Angeles, CA 90089, USA.
[b] The Bridge@USC, University of Southern California, Los Angeles, CA 90089, USA.
[c] Department of Chemistry and Biochemistry, University of California, San Diego, La Jolla, CA 92093, USA.

* Email: prezhdo@usc.edu, jsfig@ucsd.edu, brutchey@usc.edu

Experimental

Materials. Red phosphorus (amorphous, Spectrum Chemical) was used as received. Grey arsenic (99%, Strem) and antimony (99.5%, Strem) were dried under vacuum overnight prior to use. Ethylenediamine (en, ≥99.5%, Sigma-Aldrich) and ethanethiol (ET, ≥98%, Sigma-Aldrich) were dried for 12 h over 3 Å molecular sieves, and oxygen was removed with three freeze-pump-thaw cycles using standard Schlenk techniques. *N*,*N*-dimethylformamide (DMF) was used as received (AR, Macron). *WARNING:* White phosphorus is highly flammable and pyrophoric upon contact with air; it is also toxic.

Red phosphorus dissolution and recovery. Reactions were performed under a nitrogen atmosphere using standard air-free techniques. A typical 20 mg/mL solution was prepared by combining 60 mg (1.9 mmol) red phosphorus with 0.6 mL (8.1 mmol) ET and 2.4 mL (35.9 mmol) en. The 1:4 vol/vol ratio of ET to en was chosen based on precedent of thiol-amine dissolution of bulk solids, which generally operate in the range of 1:4–1:10 vol/vol of thiol to amine. This reaction was stirred at *ca.* 400 rpm, heated to 55 ˚C, and allowed to react for 48 h. The resulting solution was filtered using a 45 μm PTFE syringe filter. A lower concentration reaction was performed using the same method with 3.75 mg (0.04 mmol) red phosphorus.

To recover the red phosphorus, a 1 mL volume of the filtered solution was drop cast onto a glass borosilicate microscope slide and heated to 320 ˚C under flowing N_2 for 30 min before allowing it to cool naturally to room temperature.

Analogous reactions with P_4 and other elemental pnictogens. Reactions with P_4 were performed in an Ar-filled glove box. 5 mg (0.04 mmol) P_4 was added to a mixture of 0.2 mL ET and 0.8 mL en and stirred at 55 ˚C for 48 h. Dissolution was observed within minutes.

Grey arsenic and elemental antimony were dissolved using a fully analogous method to red phosphorus dissolution.

Characterization. *Nuclear magnetic resonance (NMR):* Solution ^{31}P NMR spectra of dissolved red phosphorus were acquired on a Varian 600 NMR spectrometer. All samples in ET and en were diluted with 25 vol% acetonitrile-d_3 as the NMR lock solvent in air-free J. Young tubes. The sample remained fully soluble. An NMR sample without was acetonitrile-d_3 was analyzed and showed no difference in the ^{31}P NMR spectrum, indicating no secondary reaction with the deuterated solvent. ^{31}P NMR spectra were taken at 243 MHz using two windows of -200-10 ppm and -10-200 ppm for 1024 scans each at a 45˚ pulse angle with a 1 s relaxation delay. A ^{31}P NMR spectrum of the full window was taken to ensure no peaks were the result of spectral aliasing or folding. ^{31}P NMR spectra of dissolved P_4 were acquired on a 300 MHz Bruker NMR with a single window of -300 to 300 ppm for 1024 scans at a 30˚ pulse angle with a 1 s relaxation delay. *Electrospray ionization mass spectrometry (ESI-MS):* Mass spectra were collected on an Agilent 6545 qToF instrument equipped with dual AJS electrospray ionization source operating in negative ion mode with following ionization parameters: capillary voltage 4.0 kV, nozzle voltage 1.0 kV, nitrogen was applied as a nebulizer gas 35 psi, sheath gas 8 L/min, 250 °C, dry gas 10 L/min, 275 °C, and collision gas. For external calibration and tuning a low-concentration tuning mix solution by Agilent Technologies was utilized at 10:1 further dilution. Spectra were recorded in *m/z* 50-1500 range at 1 Hz. The samples were prepared by 100× dilutions in DMF and injected through the main nebulizer using a syringe pump, fitted with a 500 µL Hamilton syringe (1750RN) at 5 µL/min flow rate. *Gas chromatography mass spectrometry (GC-MS):* Measurements were performed with an Agilent 7890 GC system equipped with an Agilent 7250 qToF mass detector (electron ionization, 70 eV) and an HP-5MS column (30 m × 0.25 mm × 0.25 µm film) using He as a carrier gas at a flow of 1.2 mL min^{-1}. The following temperature program was used in the GC-MS measurements: initial temperature: 40 °C, hold for 1 min, then 32 °C min^{-1} to 300 °C and hold for 3.875 min. Nitrogen was used as a collision gas. All the MS spectra were recorded at 5 Hz. Samples were prepared by dilution in 1 mL of dichloromethane and the red phosphorus solution was diluted to 0.1 mg/mL phosphorus content. *Thermogravimetric analysis (TGA):* Data were obtained on a TA Instruments TGA-Q50. The samples were prepared by putting a few drops of the filtered red phosphorus solution into an alumina TGA pan, purging under N_2 for 30 min, then heating to 100 ˚C for 10 min under flowing N_2. The pan was then transferred to the TGA furnace where it was purged under flowing N_2 for 30 min. The TGA was ramped at 10 ˚C/min to 500 ˚C and held at 500 ˚C for 10 min. *Powder X-ray diffraction (XRD):* XRD patterns were acquired on a Rigaku Ultima IV diffractometer operating at 40 mA and 44 kV with a Cu K^{α} X-ray source (λ = 1.5406 Å). Diffraction patterns were collected from 10 to 70˚ 2θ at 1 dpm. *Scanning electron microscopy-energy dispersive X-ray spectrometry (SEM-EDS):* SEM images and EDS maps were acquired on a Nova NanoSEM 450 Field Emission scanning electron microscope operating at 20 kV at 500× magnification. A minimum of 5 million counts were obtained for EDS mapping. *Fourier-transform infrared spectroscopy (FT-IR):* FT-IR spectra were collected on an Agilent Diamond ATR IR spectrometer from 400-4000 cm^{-1}. *Raman spectroscopy:* Raman spectra were obtained on a Horiba Raman Infrared Microscope, using a 532 nm laser at 50% power from 0-

3000 cm^{-1}, for five 5 s scans. *Electrolytic conductivity:* Conductivity measurements were performed using an Epsilon Basi potentiostat with silver wire as reference and working electrodes. *Ultraviolet-visible spectroscopy (UV-vis):* UV-vis absorption spectra were acquired on a Perkin-Elmer UV-vis spectrophotometer using a 1-cm path length quartz cuvette placed in a 15-cm integrating sphere. The data was collected from 250-800 nm with halogen and deuterium lamp sources.

Computational methodology. The geometry optimizations were performed at the density functional theory (DFT) level, whereas the excited states and absorption spectra derived from vertical excitations were computed using the time-dependent DFT (TD-DFT) method through the Gaussian16 software package.[1] The counterions were replaced with ammonium to decrease the computational cost. The geometries of all structures were optimized with the PBE functional and 6-311++g(d,p) basis set level of theory[2-5]. In contrast, the excited state calculations were performed with the more accurate ωB97XD hybrid functional,[6] which employs a version of Grimme's D2 dispersion correction model and the 6-311++g(d,p) basis set. ωB97XD was selected to achieve an accurate description of both localized and delocalized states. The solvent was modeled using the conductor polarizable continuum model (C-PCM),[7,8] using the dielectric constant $\varepsilon = 37.219$ corresponding to *N*,*N*-dimethylformamide. While an equilibrium solvation model was used during the geometry optimization, a non-equilibrium solvation model (vertical excitations) was employed for the excited state calculations.

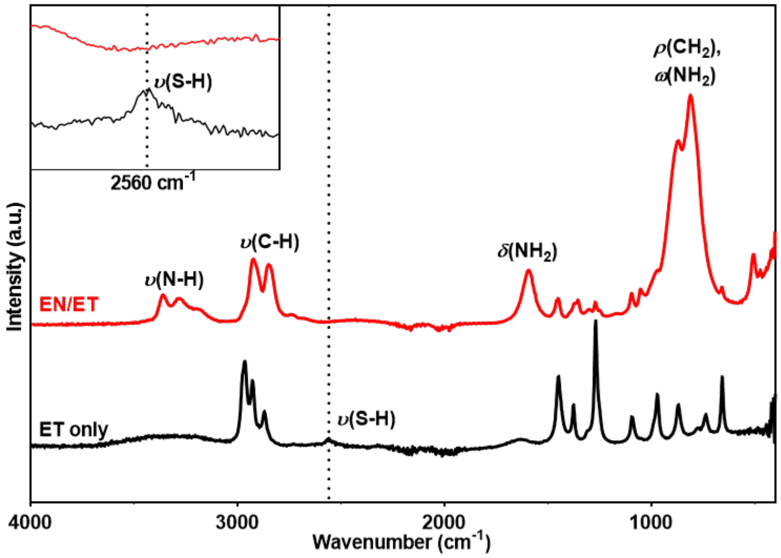

Figure S1. FT-IR spectra of ethanethiol (black) and ethanethiol-ethylenediamine mixture (red). Inset shows the loss of the weak thiol *ν*(S–H) at 2560 cm^{-1}.

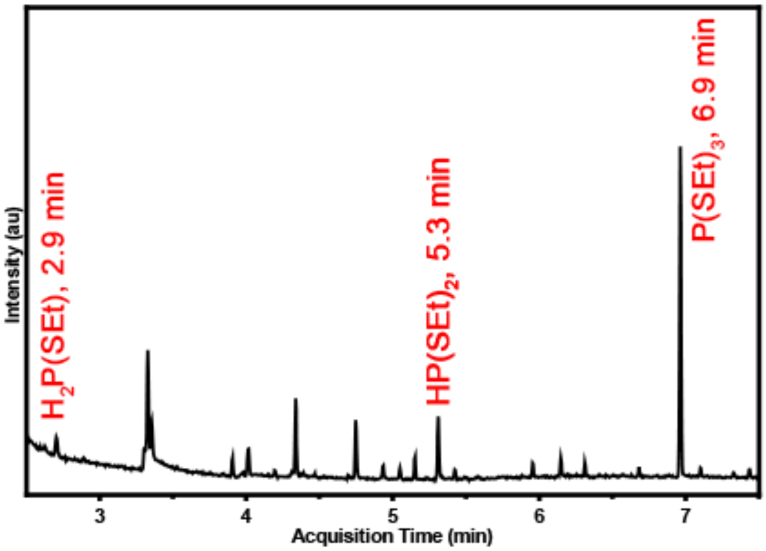

Figure S2. GC-MS of red phosphorus solution showing elution of $H_xP(SEt)_{3-x}$ species, where x = 0-2.

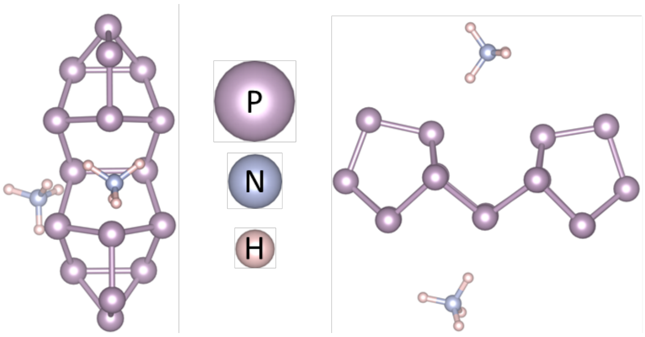

Figure S3. Charge neutral model of $P_{16}(NH_4)_2$.

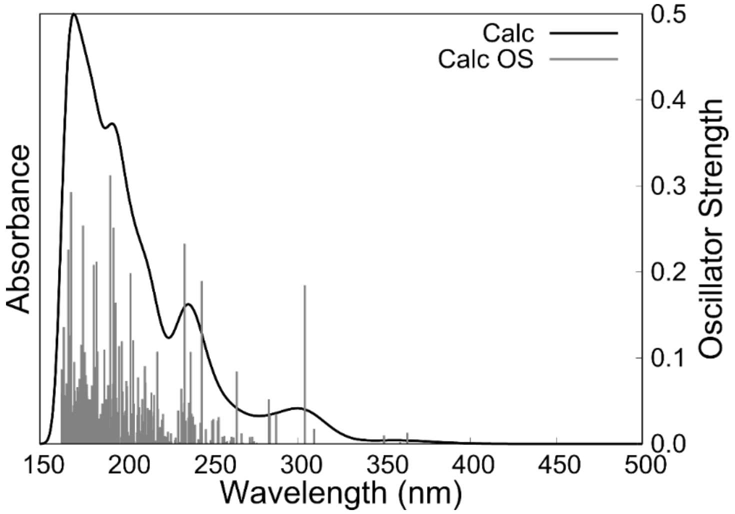

Figure S4. Absorption spectrum of $P_{16}(NH_4)_2$ with CPCM solvation model (solvent = *N*,*N*-dimethylformamide). The calculated oscillator strengths (OC) for the first 300 excited states are shown by the vertical sticks with the scale shown on the right. The continuous spectrum is obtained by broadening each line in the stick spectrum.

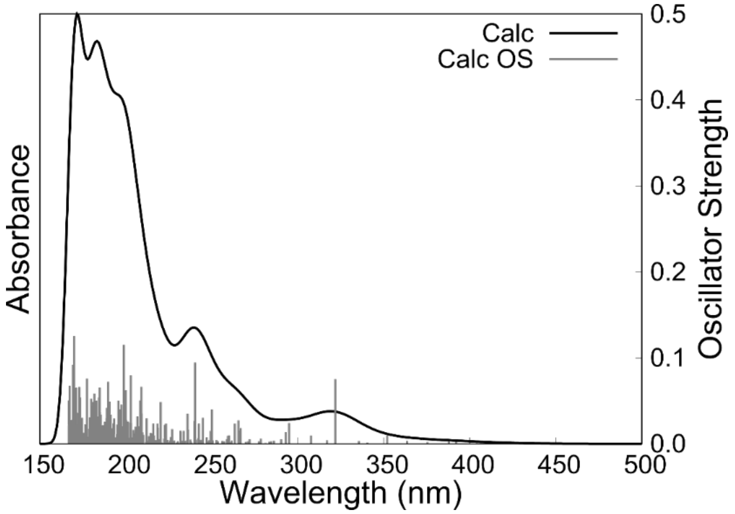

Figure S5. Absorption spectrum of $P_{16}(NH_4)_2$ in vacuum (black, left scale) and calculated oscillator strength (OS) for the first 300 excited states (gray sticks, right scale).

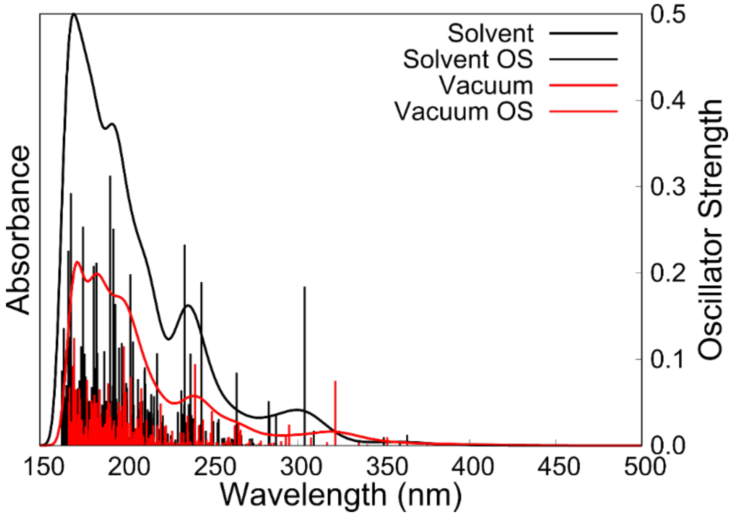

Figure S6. Comparison of the absorption spectra of $P_{16}(NH_4)_2$ in vacuum (black, left scale) and with CPCM solvation (solvent = *N*,*N*-dimethylformamide) (red, left scale). The calculated oscillator strengths (OC) for the first 300 excited states are shown by the corresponding vertical sticks (right scale). Solvation reduces the intensity because it decreases the transition dipole moments of the species.

Excited State Transition	Vacuum		Solvent	
	NTO Occupied	NTO Unoccupied	NTO Occupied	NTO Unoccupied
3	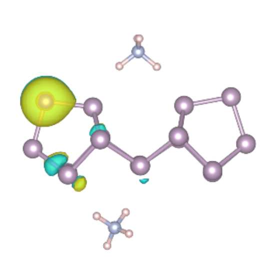	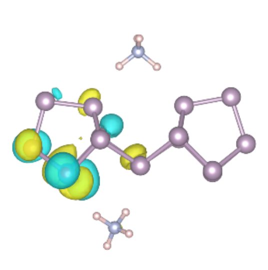	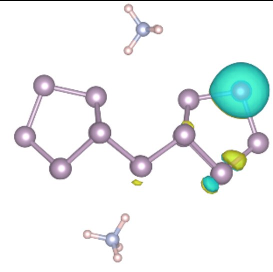	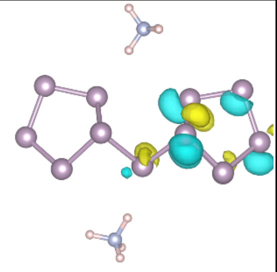
	Energy: 392.16 nm<br>Oscillator Strength: 0.0022		Energy: 363.65 nm<br>Oscillator Strength: 0.0127	
	Main MOs 130→133 (NTO pair 90%)		Main MOs 131→135 (NTO pair 88%)	
10	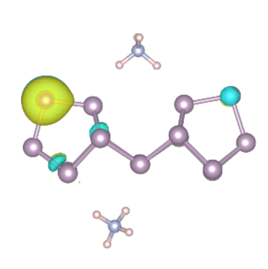	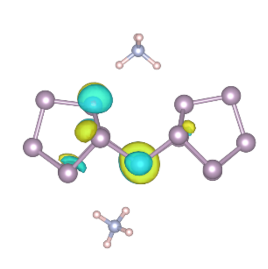	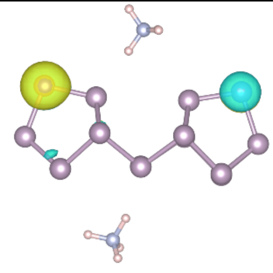	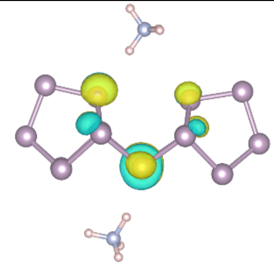
	Energy: 321.74 nm<br>Oscillator Strength: 0.0750		Energy: 303.95 nm<br>Oscillator Strength: 0.1843	
	Main MOs 131→137 (NTO pair 83%)		Main MOs 130→136 (NTO pair 73%)	
53	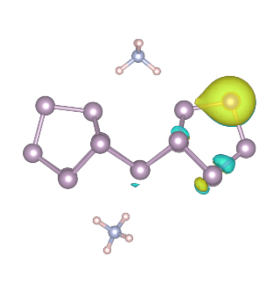	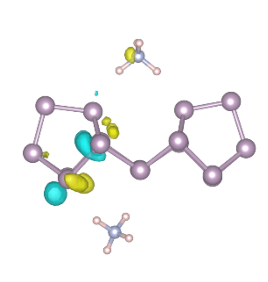	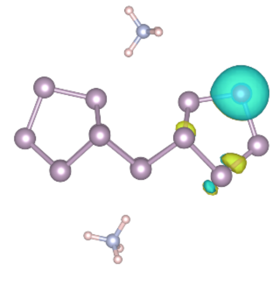	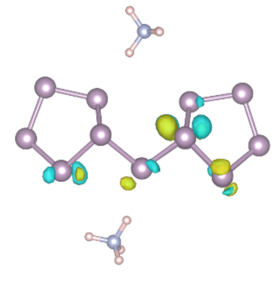
	Energy: 240.74 nm<br>Oscillator Strength: 0.0048		Energy: 234.24 nm<br>Oscillator Strength: 0.2329	
	Main MOs 131→152 (NTO pair 40%)		Main MOs 130→146 (NTO pair 47%)	

Figure S7. Natural transition orbital (NTO) pairs for three major electronic transitions observed in the solution calculation (right) compared to the corresponding transitions of the vacuum model (left). Isovalue = 0.05 eV. The percent contribution is given for each NTO pair and the highest contributing MO pair.

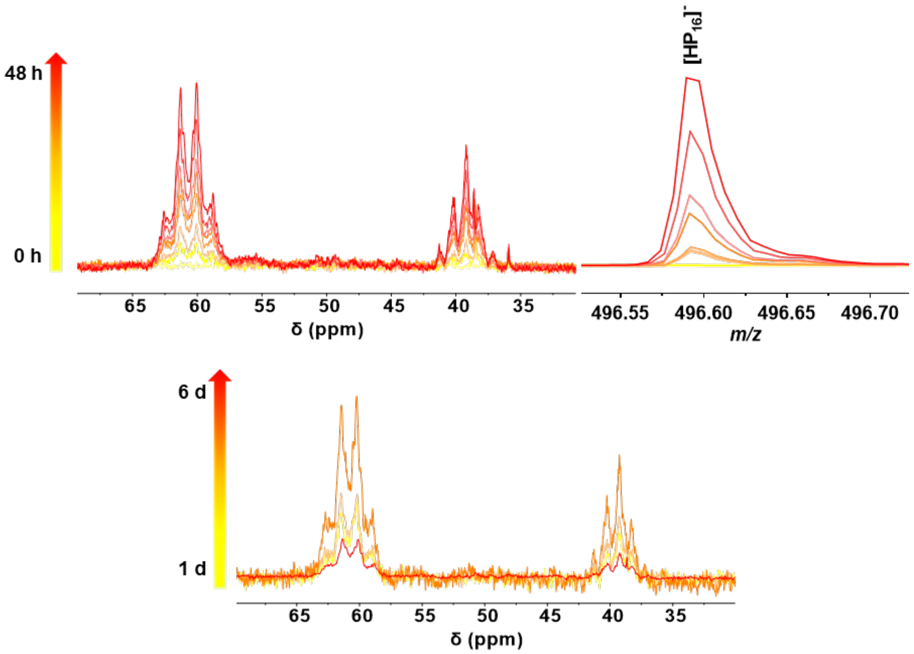

Figure S8. Time studies of the formation of P_{16}^{2-} in ET and en. (top) Solution $^{31}P\{^1H\}$ NMR spectra (left) and ESI-(–)MS (right) of P_{16}^{2-} peaks over the first 48 h of reaction. (bottom) Solution $^{31}P\{^1H\}$ NMR spectra of P_{16}^{2-} peaks over 6 d.

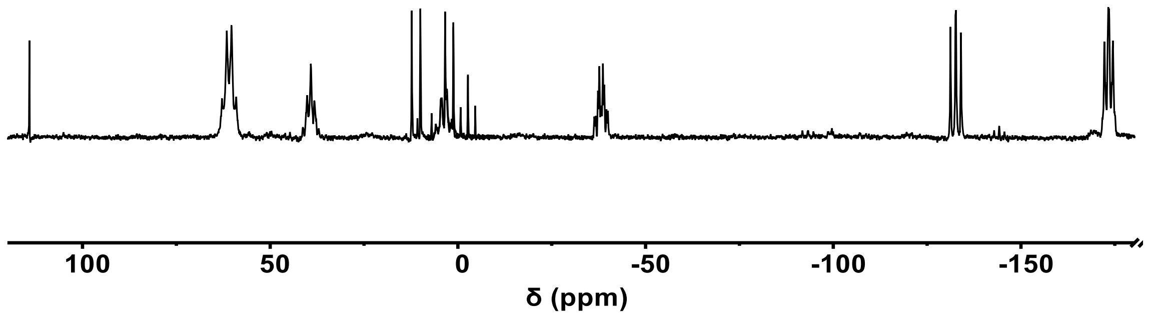

Figure S9. Proton coupled solution ^{31}P NMR spectrum of red phosphorus solution in acetonitrile-*d*3.

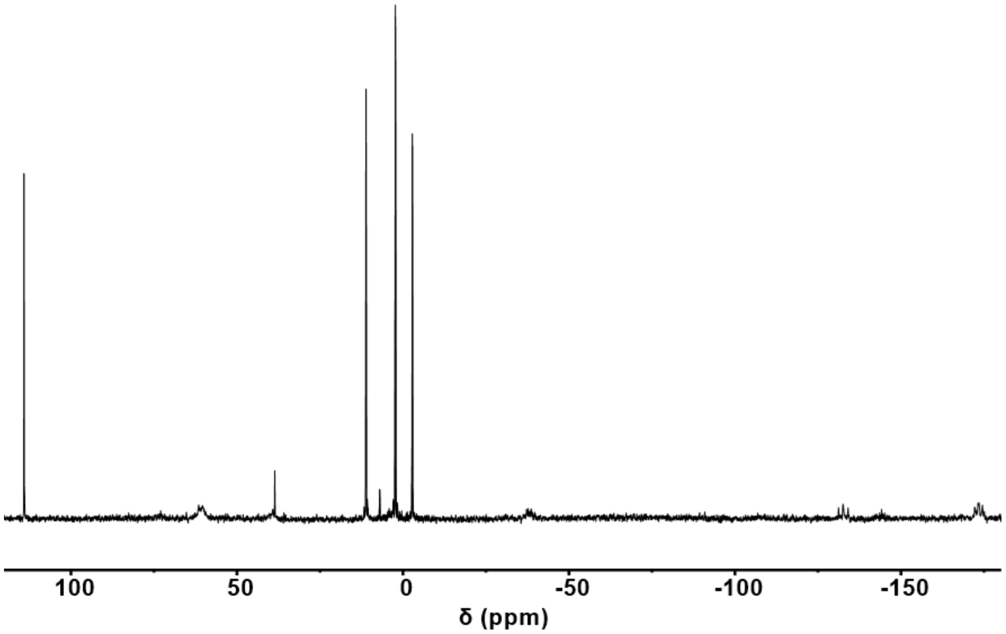

Figure S10. Solution ^{31}P NMR spectrum of low concentration (5 mg/mL) red phosphorus solution in acetonitrile-d_3

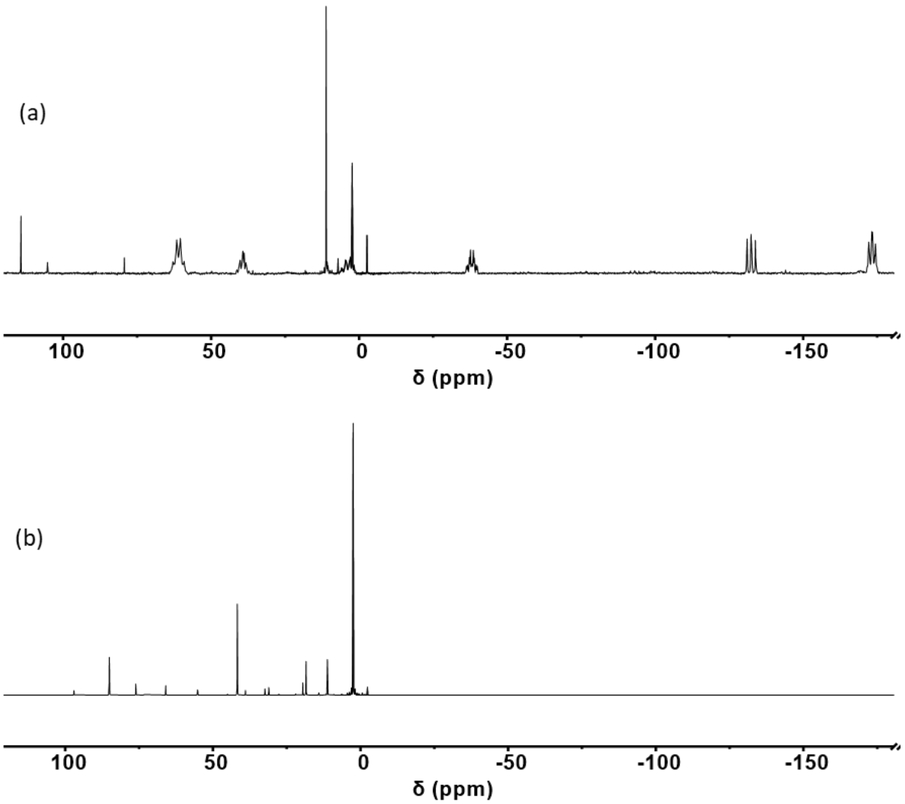

Figure S11. Solution $^{31}P\{^1H\}$ NMR spectra of red phosphorus solution in acetonitrile-d_3 (a) immediately after 2 d air-free reaction and (b) after 5 d of air exposure.

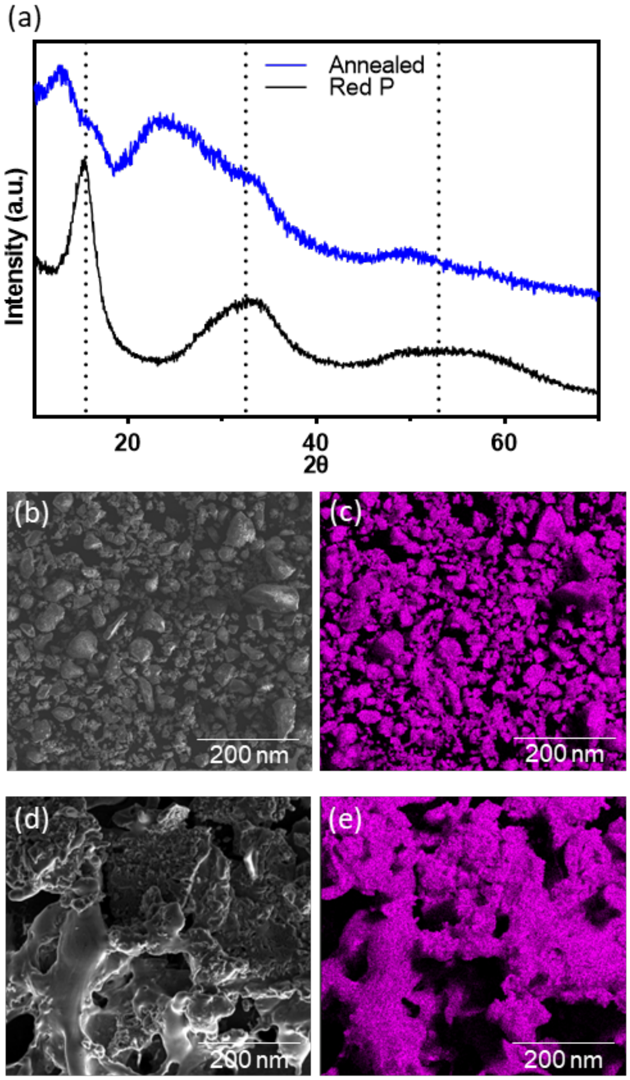

Figure S12. Comparison of as-purchased red phosphorus to red phosphorus recovered from solution after annealing. (a) Powder XRD pattern of as-purchased red phosphorus (black) and recovered red phosphorus after annealing (blue). Red phosphorus diffraction peaks are denoted by vertical dotted lines. SEM images of (b) as-purchased red phosphorus and (d) recovered red phosphorus after annealing. SEM-EDS mapping of phosphorus in (c) as-purchased red phosphorus and (e) recovered red phosphorus after annealing.

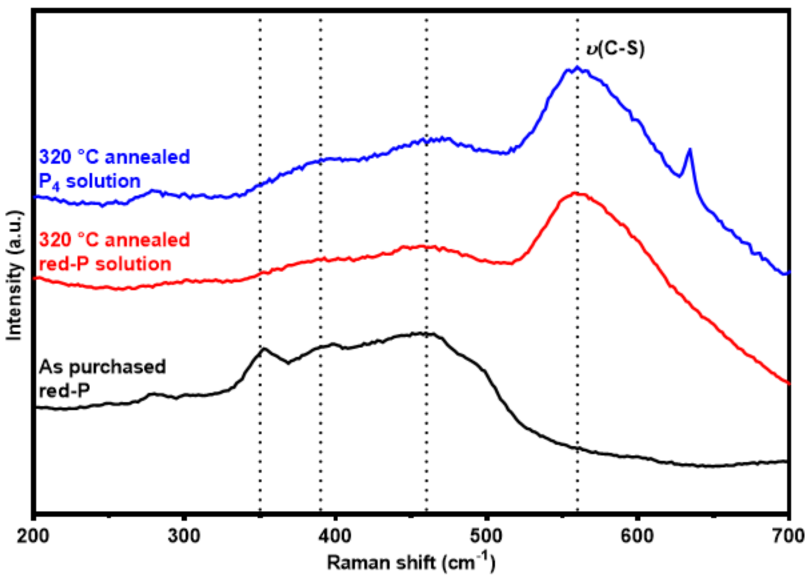

Figure S13. Raman spectra of as-purchased red phosphorus (black) and recovered amorphous red phosphorus after annealing reaction mixtures from dissolved red phosphorus (red) and P_4 (blue) at 320 ˚C. Red phosphorus bands and the ν(C–S) band are denoted by vertical dotted lines.

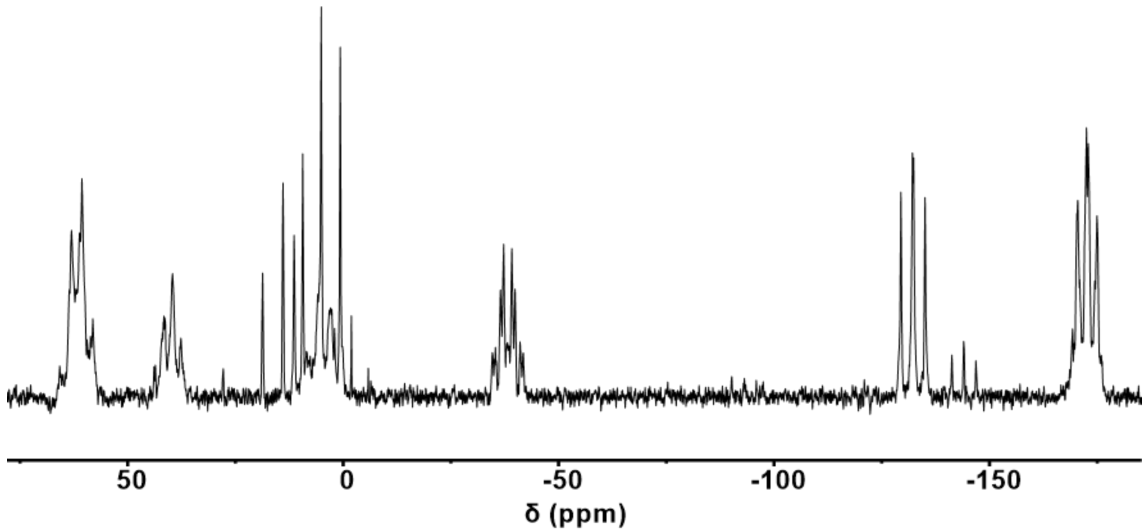

Figure S14. Proton coupled solution ^{31}P NMR spectrum of P_4 reacted with ET and en in acetonitrile-d_3.

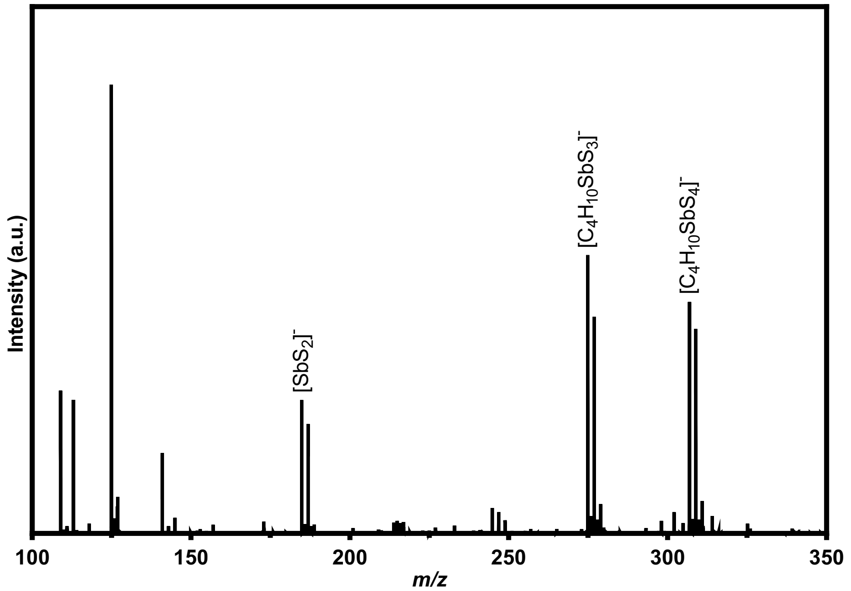

Figure S15. Negative mode ESI mass spectrum of the reaction of elemental antimony with ET and en, diluted 100× volumetrically in DMF.

References:

1. Frisch, M. J.; Trucks, G. W.; Schlegel, H. B.; Scuseria, G. E.; Robb, M. A.; Cheeseman, J. R.; Scalmani, G.; Barone, V.; Petersson, G. A.; Nakatsuji, H.; Li, X.; Caricato, M.; Marenich, A. V.; Bloino, J.; Janesko, B. G.; Gomperts, R.; Mennucci, B.; Hratchian, H. P.; Ortiz, J. V.; Izmaylov, A. F.; Sonnenberg, J. L.; Williams-Young, D.; Ding, F.; Lipparini, F.; Egidi, F.; Goings, J.; Peng, B.; Petrone, A.; Henderson, T.; Ranasinghe, D.; Zakrzewski, V. G.; Gao, J.; Rega, N.; Zheng, G.; Liang, W.; Hada, M.; Ehara, M.; Toyota, K.; Fukuda, R.; Hasegawa, J.; Ishida, M.; Nakajima, T.; Honda, Y.; Kitao, O.; Nakai, H.; Vreven, T.; Throssell, K.; Montgomery, J. A., Jr.; Peralta, J. E.; Ogliaro, F.; Bearpark, M. J.; Heyd, J. J.; Brothers, E. N.; Kudin, K. N.; Staroverov, V. N.; Keith, T. A.; Kobayashi, R.; Normand, J.; Raghavachari, K.; Rendell, A. P.; Burant, J. C.; Iyengar, S. S.; Tomasi, J.; Cossi, M.; Millam, J. M.; Klene, M.; Adamo, C.; Cammi, R.; Ochterski, J. W.; Martin, R. L.; Morokuma, K.; Farkas, O.; Foresman, J. B.; Fox, D. J. Gaussian 16, Rev. C.01, Gaussian, Inc., Wallingford CT, 2016.
2. Perdew, J. P.; Burke, K.; Ernzerhof, M. Generalized Gradient Approximation Made Simple. *Phys. Rev. Lett.* **1996**, *77*, 3865–3868.
3. Perdew, J. P.; Burke, K.; Wang, Y. Generalized Gradient Approximation for the Exchange-Correlation Hole of a Many-Electron System. *Phys. Rev. B* **1996**, *54*, 16533–16539.
4. Ditchfield, R.; Hehre, W. J.; Pople, J. A. Self-Consistent Molecular-Orbital Methods. IX. An Extended Gaussian-Type Basis for Molecular-Orbital Studies of Organic Molecules. *J. Chem. Phys.* **2003**, *54*, 724.
5. Hehre, W. J.; Ditchfield, R.; Pople, J. A. Self-Consistent Molecular Orbital Methods. XII. Further Extensions of Gaussian—Type Basis Sets for Use in Molecular Orbital Studies of Organic Molecules. *J. Chem. Phys.* **2003**, *56*, 2257.

6. Chai, J.-D.; Head-Gordon, M. Long-Range Corrected Hybrid Density Functionals with Damped Atom–Atom Dispersion Corrections. *Phys. Chem. Chem. Phys.* **2008**, *10*, 6615–6620.
7. Mei, J.; Wang, F.; Wang, Y.; Tian, C.; Liu, H.; Zhao, D. Energy Transfer Assisted Solvent Effects on $CsPbBr_3$ Quantum Dots. *J. Mater. Chem. C* **2017**, *5*, 11076–11082.
8. Barone, V.; Cossi, M. Quantum Calculation of Molecular Energies and Energy Gradients in Solution by a Conductor Solvent Model. *J. Phys. Chem. A* **1998**, *102*, 1995–2001.
